# Machine learning-based prediction of 90-day prognosis and in-hospital mortality in hemorrhagic stroke patients

**DOI:** 10.1038/s41598-025-90944-x

**Published:** 2025-05-09

**Authors:** Ahmad A. Abujaber, Ibrahem Albalkhi, Yahia Imam, Said Yaseen, Abdulqadir J. Nashwan, Naveed Akhtar, Ibrahim M. Alkhawaldeh

**Affiliations:** 1 Nursing Department, Hamad Medical Corporation, P.O. Box 3050, Doha, Qatar; 2 College of Medicine, Alfaisal University, Riyadh, Saudi Arabia; 3 Department of Neuroradiology, Great Ormond Street Hospital NHS Foundation Trust, Great Ormond St, London, WC1N 3JH UK; 4 Neurology Section, Neuroscience Institute, Hamad Medical Corporation, Doha, Qatar; 5 School of Medicine, Jordan University of Science and Technology, Irbid, Jordan; 6 Faculty of Medicine, Mutah University, Al-Karak, Jordan

**Keywords:** Hemorrhagic stroke, Stroke registry, Prognosis, Mortality, Machine learning, Neuroscience, Neurology, Stroke

## Abstract

This study aims to predict hemorrhagic stroke outcomes, including 90-day prognosis and in-hospital mortality, using machine learning models and SHapley Additive exPlanations (SHAP) analysis. Data were collected from a national Stroke Registry from January 2014 to July 2022. Various predictive factors were considered, such as stroke severity at presentation, patient demographics, laboratory results, admission location, and other clinical features. Random forest, logistic regression, XGboost, support vector machines, and decision trees were trained and evaluated. SHAP analysis was conducted to identify key predictors. The RF model demonstrated superior performance in predicting prognosis, while LR was more effective in predicting in-hospital mortality. The National Institute of Health Stroke Score (NIHSS) and admission location were key predictors. Despite its limitations, this research underscores the importance of advancing stroke registries and emphasizes the necessity for comprehensive external validation of predictive models. Furthermore, it demonstrates the importance of initial stroke severity in influencing patient outcomes and highlights the significance of admission to stroke units in reducing poor outcomes. This may help shape interventions to enhance stroke center capacities and influence strategic policies. This study contributes towards developing more precise predictive models for hemorrhagic stroke outcomes, potentially impacting clinical practice and optimizing resource allocation significantly.

## Introduction

Stroke is a major public health issue, impacting 13.7 million people annually and causing 5.5 million deaths^1^, as well as significant long-term disability^2^. While ischemic strokes are more frequent^3^, hemorrhagic strokes are more severe, with in-hospital mortality rates of 20–30% and up to 80% of survivors requiring long-term care^4^.

Various risk factors, including diabetes, COPD, high blood pressure, smoking, diet, and physical activity, are associated with different types of strokes^4^. However, certain risk factors are more closely linked to specific stroke subtypes. Hypertension, for instance, increases the risk of severe outcomes in hemorrhagic strokes compared to ischemic strokes^5^, while diabetes is linked to poorer outcomes in ischemic strokes^6^. A study in Uganda found that individuals recovering from hemorrhagic strokes experienced more severe impairments within 30 days post-discharge compared to those recovering from ischemic strokes, as measured by the modified Rankin scale (mRS)^7^.

Hemorrhagic strokes are more common in males, accounting for 10–20% of annual stroke cases^8^. A study from 2000 to 2019 found that while both sexes with intracerebral hemorrhage (ICH) saw declining favorable outcomes, only females improved in unfavorable outcomes and mortality rates. For subarachnoid hemorrhage (SAH), both genders showed improvements in outcomes and mortality^9^. Beyond physical effects, hemorrhagic strokes also lead to psychological issues like anxiety, depression, fatigue, and sleep disturbances, particularly in patients with higher mRS scores, as shown by research from Sarah Ecker and colleagues^10^.

Modern healthcare focuses on preventing adverse outcomes by customizing patient care plans. This approach requires significant investment in improving clinicians’ ability to predict outcomes, allowing personalized care that enhances patient and family quality of life^11^. Scholars have worked extensively to predict stroke outcomes, including short- and long-term prognosis, disabilities, and mortality, using variables like patient demographics, health conditions, lab results, stroke severity, and hospital-related factors. For instance, Wang et al. identified age, hemorrhage size, location, and the presence of diabetes and hypertension as key factors in predicting a one-month hemorrhagic stroke prognosis^12^. Matsuo and colleagues noted that lifestyle habits, such as smoking, impact recovery, with non-smokers more likely to have favorable outcomes^13^. Research consistently shows that hemorrhagic stroke prognosis varies by location, with strokes in the cerebral lobes generally having better outcomes than those in deeper brain structures like the thalamus^14^.

Some researchers have used established tools to predict stroke prognosis, including mortality. Moon and colleagues employed the Acute Physiology and Chronic Health Evaluation II (APACHE II), a tool for predicting mortality in critically ill patients, which effectively predicted both ischemic and hemorrhagic stroke mortality with an AUC of 0.80^15^. Abdelghany and team used the modified Stroke subtype, Oxfordshire Community Stroke Project Classification, Age, and pre-stroke Rankin score (mSOAR) to predict 7-day in-hospital mortality and post-stroke disability, achieving AUC values between 0.8 and 0.82^16^.

The use of machine learning in medical research has significantly expanded, particularly in screening, diagnosis, and prognosis. Machine learning algorithms show great promise in predicting stroke outcomes, often matching, or surpassing traditional methods in accuracy for 90-day prognosis and mortality for both ischemic and hemorrhagic strokes. Recent advancements in interpretable machine learning techniques have also improved the accuracy of predicting functional outcomes at discharge. Building on this potential, our study seeks to leverage these models to predict in-hospital mortality and 90-day post-discharge prognosis for hemorrhagic stroke patients, using the mRS as the evaluation metric, thus laying the groundwork for a more detailed methodological approach.

## Methods

### Study population

We collected data from the Stroke Registry at Hamad General Hospital (HGH) from January 2014 to July 2022. The dataset includes all individuals aged 18 years and older who were admitted to HGH with a primary diagnosis of stroke. Since the establishment of the stroke registry in Qatar until July 2022, a total of 15,859 patients have sought specialized stroke treatment at the hospital. This figure encompasses patients diagnosed with ischemic and hemorrhagic strokes, transient ischemic attacks (TIAs), and stroke mimics. However, our study specifically focuses on patients diagnosed with hemorrhagic strokes, with all other conditions excluded.

### Baseline variables

The collected data included a wide range of patient details, covering demographics, hemodynamic measurements upon admission (e.g., heart rate (HR) and blood pressure (BP)), laboratory results, factors that contribute to stroke risk, pre-existing medical conditions, admission locations, and hospitalization outcomes (e.g., length of stay (LOS), and hospital-acquired infections (e.g., Urinary Tract Infection and Pneumonia). Stroke severity at admission was assessed using the National Institute of Health Stroke Score (NIHSS).

In terms of ethnicity, patients were grouped into five specific categories based on their reported nationality: Qatari, Middle East and North Africa (MENA) region, South Asia region, Southeast Asia region (defined according to the United Nations geo-scheme), and all other nationalities were combined into “others” category. It is important to note the distinct classification for Qatari patients, which was implemented to facilitate meaningful comparisons. This categorization considers the unique demographic makeup of the country, where expatriates constitute a sizable portion of the population. This methodology has consistently been used in prior research examining stroke in Qatar. All relevant risk factors, including pre-existing medical conditions and smoking history, were documented during the patient’s hospital stay and cross-verified by stroke registry personnel through electronic medical records. Twenty-nine variables were used for predicting the prognosis (mRS-90) and twenty-eight were used to predict the in-hospital mortality. The details are presented in Table 1.

**Table 1:** Statistical characteristics of the collected stroke dataset.

	90-day modified Rankin Score			In-hospital Mortality				
Variable	Feature	Favorable (mRS ≤ 2)	Unfavorable (mRS > 2)	Total	Feature	Alive	Died	Total
Age (years)	< Mean (51.3)	313	321	634	< Mean (50.9)	820	91	911
	≥ Mean (51.3)	202	262	464	≥ Mean (50.9)	678	68	746
	Mean ± SD (51.3 ± 13)-IQR 16			Mean ± SD (50.9 ± 13.1)-IQR 17				
Sex	1: Male	447	470	917	1: Male	1248	130	1378
	2: Female	68	113	181	2: Female	250	29	279
Ethnicity	1: Qatari	54	104	158	1: Qatari	199	25	224
	2: MENA	66	68	134	2: MENA	176	22	198
	3: South Asian	275	303	578	3: South Asian	809	76	885
	4: South-East Asian	98	96	194	4: South-East Asian	266	32	298
	5: Other	22	12	34	5: Other	48	4	52
Mode of Arrival	1: Ambulance	435	554	989	1: Ambulance	1337	143	1480
	2: Private vehicle	77	25	102	2: Private vehicle	150	16	166
	3: In-hospital	3	4	7	3: In-hospital	11	0	11
Modified Rankin Score (mRS) pre-stroke onset	< Mean (0.35)	498	483	981	< Mean (0.33)	1356	136	1492
	≥ Mean (0.35)	17	100	117	≥ Mean (0.33)	142	23	165
	Mean ± SD (0.35 ± 1.1)-IQR 0				Mean ± SD (0.33 ± 1.06)-IQR 0			
NIHSS at admission	< Mean (11.2)	424	174	598	< Mean (12)	830	14	844
	≥ Mean (11.2)	91	409	500	≥ Mean (12)	668	145	813
	Mean ± SD (11.2 ± 8.6)-IQR 15				Mean ± SD (12 ± 8.8)-IQR 16			
Body Mass Index (BMI)	1: Underweight	40	30	70	1: Underweight	130	34	164
	2: Normal weight	157	157	314	2: Normal weight	418	41	459
	3: Overweight	201	251	452	3: Overweight	611	48	659
	4: Obese	92	101	193	4: Obese	246	26	272
	5: Extremely Obese	25	44	69	5: Extremely Obese	93	10	103
Random Blood Sugar (mmol/l)	< Mean (8.7)	354	342	696	< Mean (8.8)	992	61	1053
	≥ Mean (8.7)	148	231	379	≥ Mean (8.8)	476	93	569
	Mean ± SD (8.7 ± 3.9)-IQR 3.6				Mean ± SD (8.8 ± 4)-IQR 3.8			
Systolic Blood Pressure (SBP) (mmHg)	< Mean (180)	300	275	575	< Mean (180.5)	762	86	848
	≥ Mean (180)	212	308	520	≥ Mean (180.5)	752	72	797
	Mean ± SD (180 ± 36.5)-IQR 53				Mean ± SD (180.5 ± 37.1)-IQR 54			
Diastolic Blood Pressure (DBP) (mmHg)	< Mean (106.5)	286	276	562	< Mean (106.7)	750	87	837
	≥ Mean (106.5)	226	307	533	≥ Mean (106.7)	737	71	808
	Mean ± SD (106.5 ± 24)-IQR 33				Mean ± SD (106.7 ± 24.8)-IQR 32			
Heart Rate (bpm)	< Mean (83)	262	318	580	< Mean (83.35)	793	77	870
	≥ Mean (83)	246	262	508	≥ Mean (83.35)	676	77	753
	Mean ± SD (83 ± 15.5)-IQR 19				Mean ± SD (83.35 ± 15.9)-IQR 21			
Time from onset to hospital arrival (hour)	≤ 3 h	225	358	583	≤ 3 h	776	85	861
	3–6 h	39	65	104	3–6 h	143	7	150
	6–24 h	51	47	98	6–24 h	136	6	142
	> 24 houra	146	37	183	> 24 houra	241	9	250
	Unidentified	54	76	130	Unidentified	202	52	254
Diabetes Mellitus (DM)	0: No	355	354	709	0: No	979	115	1094
	1: Yes	160	229	389	1: Yes	519	44	563
Hypertension (HTN)	0: No	79	71	150	0: No	215	61	276
	1: Yes	436	512	948	1: Yes	1283	98	1381
Dyslipidemia	0: No	346	406	752	0: No	1056	137	1193
	1: Yes	169	177	346	1: Yes	442	22	464
Prior stroke	0: No	486	528	1014	0: No	1397	147	1544
	1: Yes	29	55	84	1: Yes	101	12	113
Atrial Fibrillation (AF)	0: No	508	560	1068	0: No	1459	150	1509
	1: Yes	7	23	30	1: Yes	39	9	48
Coronary Artery Disease (CAD)	0: No	490	542	1032	0: No	1412	145	1558
	1: Yes	25	41	66	1: Yes	86	13	99
Tobacco use	0: No	438	536	974	0: No	1332	153	1485
	1: Yes	77	47	124	1: Yes	166	6	172
Known on anti-platelets pre-stroke	0: No	468	489	957	0: No	1312	137	1449
	1: Yes	47	94	141	1: Yes	186	22	208
Known on anti-coagulants pre-stroke	0: No	506	557	1063	0: No	1457	151	1608
	1: Yes	9	26	35	1: Yes	41	8	49
Known on statins pre-stroke	0: No	476	483	959	0: No	1319	140	1459
	1: Yes	39	100	139	1: Yes	179	19	198
Platelets count on admission	< Mean (260.7)	258	316	574	< Mean (258.3)	768	77	845
	≥ Mean (260.7)	239	254	493	≥ Mean (258.3)	673	67	740
	Mean ± SD (260.7 ± 78.6)-IQR 89				Mean ± SD (258.3 ± 80)-IQR 91			
Prothrombin Time on admission (seconds)	< Mean (10.9)	312	329	641	< Mean (11)	879	85	964
	≥ Mean (10.9)	172	239	411	≥ Mean (11)	533	65	598
	Mean ± SD (10.9 ± 6.4)-IQR 2.2				Mean ± SD (11 ± 6.4)-IQR 2.2			
Activated Partial Thromboplastin Time (seconds)	< Mean (27.6)	306	379	685	< Mean (27.8)	953	103	1058
	≥ Mean (27.6)	175	185	360	≥ Mean (27.8)	453	46	499
	Mean ± SD (27.6 ± 13.3)-IQR 4.4				Mean ± SD (27.8 ± 15.3)-IQR 4.4			
Admission location	1: Stroke Unit	341	191	532	1: Stroke Unit	719	5	724
	2: ICU	112	342	454	2: ICU	614	112	726
	3: Other	62	50	112	3: Other	165	42	207
Hospital acquired Pneumonia	0: No	497	423	920	0: No	1273	126	1399
	1: Yes	18	160	178	1: Yes	225	33	258
Hospital acquired Urinary Tract Infection (UTI)	0: No	503	478	981	0: No	1344	152	1496
	1: Yes	12	105	117	1: Yes	154	7	161
Length of stay (LOS) (days)	< Mean (11.2)	437	276	713				
	≥ Mean (11.2)	78	307	385				
	Mean ± SD (11.2 ± 10.2)-IQR 11.2							
90-day mRS	0: Favorable (≤ 2) 1: Unfavorable (> 2)	515	583	1098				
In-hospital Mortality					0: Alive 2: Died in-hospital	1498	159	1657

### Outcome measures

In this study, we focused on two key outcome variables: the 90-day post-hospital discharge prognosis assessed by the modified Rankin Scale (mRS-90) and in-hospital mortality among patients with hemorrhagic stroke. For the mRS-90, which was collected during the follow-up visit 90 days after discharge, we simplified it into a binary variable to enhance the model’s parsimony and to facilitate the interpretation of the output. An mRS score of ≤ 2 was categorized as a favorable prognosis, indicating a good prognostic outcome, while an mRS score of > 2 was categorized as an unfavorable prognosis, indicating a poorer outcome. As for in-hospital mortality, patients who passed away during their hospitalization following the stroke incident were considered deceased, while those who were discharged after their admission for hemorrhagic stroke were categorized as survivors.

### Inclusion/exclusion criteria

**Fig. 1:**
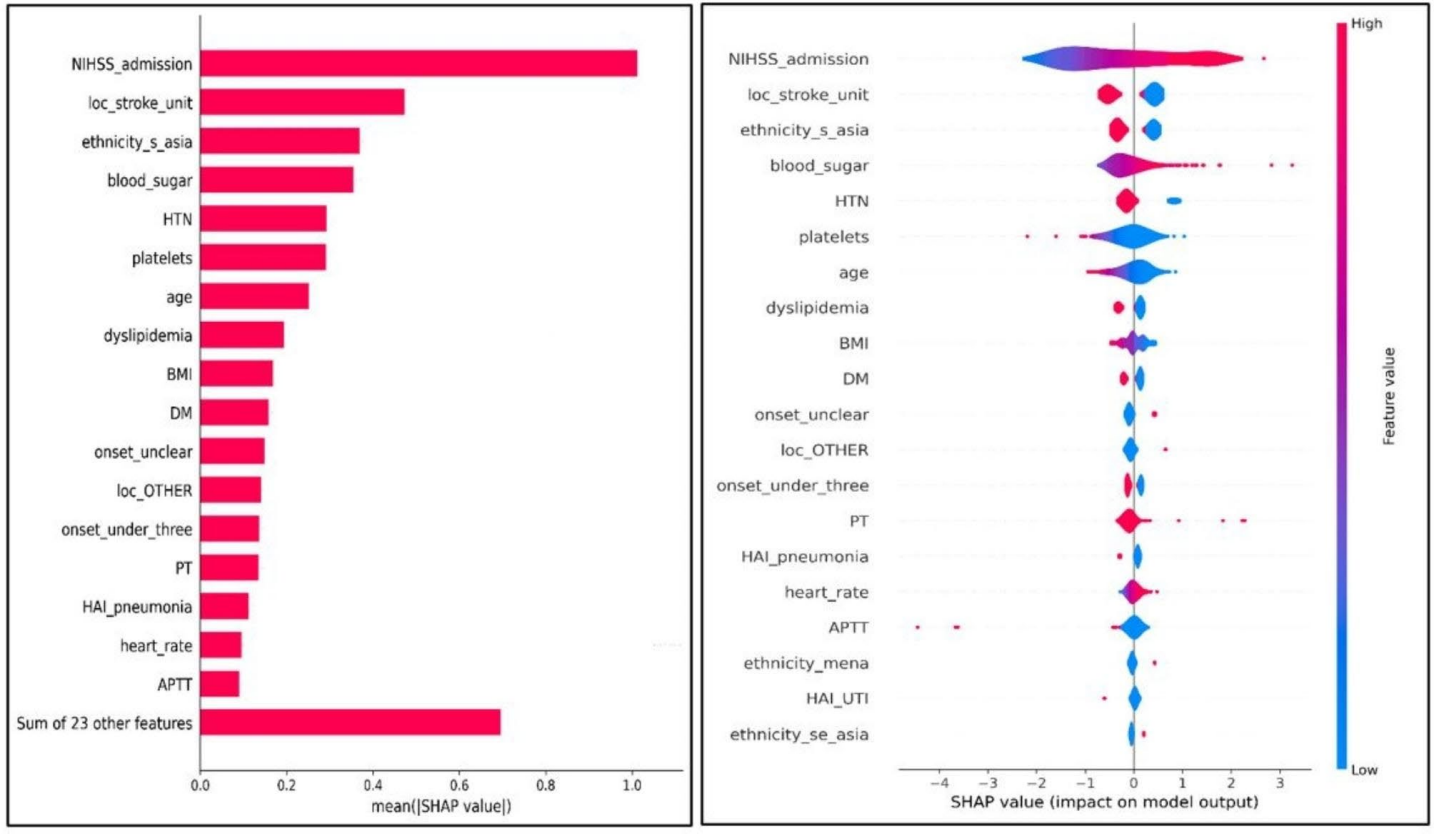
Data inclusion and exclusion procedure.

This study encompassed all adult patients aged 18 years or older who received a diagnosis of hemorrhagic stroke. From the initial cohort of 15,859 patients, 1657 patients who were diagnosed with hemorrhagic stroke were 1657, and data on functional outcomes (mRS-90) were available for 1098 patients. Figure 1 summarizes the data inclusion/exclusion procedure.

### Handling missing data and class imbalance

Multiple Imputations using the Chained Equations (MICE) technique to generate data imputations^30^ were employed in cases of missing data. The missing data included activated partial thromboplastin time (APTT) at 6.2%, prothrombin time (PT) at 5.7%; platelet counts at 4.3%, heart rate at 2.1%, blood sugar levels at 2.1%, and blood pressure at 0.7%. The cohort exhibited a mortality rate of 9.6%, resulting in concerns about class imbalance. Class weighting to mitigate the imbalance was utilized. Specifically, class weights inversely proportional to class frequencies were assigned, giving more weight to the minority class (patients who died) to enhance their influence during training. It is important to note that the mRS-90 (favorable vs. unfavorable) had an even distribution of classes.

### Model training and evaluation

The dataset was divided into a training set (80%) and a validation set (20%) using stratified random sampling. We constructed and fine-tuned our models using the training data and then assessed their performance using the validation data. We trained five different machine learning models, including extreme gradient boosting (XGBoost), random forest (RF), support vector machine (SVM), logistic regression (LR), and decision trees (DT).

To gauge the effectiveness of these models, we employed multiple classification metrics, which included accuracy, precision, specificity, recall, F1-score, area under the receiver operating characteristic curve (AUC), Matthew’s correlation coefficient (MCC), log loss, and Brier score. These metrics offer insights into how well the models can accurately classify positive and negative instances while considering the class imbalance. The model with the highest F1-score will be selected as the primary model for subsequent external and temporal validation.

We also utilized the SHAP (SHapley Additive exPlanations) library, a powerful tool for interpreting machine learning models’ predictions. This tool generates individual-level feature importance scores known as SHAP values, which quantify the contribution of each feature to a specific prediction outcome.

### Ethics and inclusion statement

This study adhered to ethical standards by including local researchers throughout all phases of the research process, including study design, implementation, data ownership, intellectual property, and authorship of publications. The involvement of local partners ensured that the research remained locally relevant and addressed context-specific concerns. Roles and responsibilities among all collaborators were agreed upon at the outset of the project, and capacity-building initiatives for local researchers were discussed and incorporated into the research framework. The research adhered to all applicable local regulations and would not have been restricted or prohibited in the setting of the researchers. No specific exceptions were required for this study, as it was in complete agreement with local stakeholders. The research received approval from the Institutional Research Board (IRB) at Hamad Medical Corporation, Qatar, with reference number MRC-01-22-594, in compliance with ethical guidelines. No aspect of the research posed a risk of stigmatization, incrimination, or discrimination for the participants. Provisions were made to ensure the safety and well-being of all participants. Additionally, risk management plans were implemented to protect the researchers’ health, safety, and security. No biological materials, cultural artifacts, or associated traditional knowledge were transferred out of the country. Finally, local and regional research relevant to the study was duly considered and appropriately cited to reflect the contributions of the regional academic community.

## Results

### Baseline characteristics

Table 1 presents the study population’s characteristics for both outcome measures. Obviously, 53% of the patients presented with an unfavorable mRS of 90 days post-discharge, while 9.5% had in-hospital mortality.

### Model evaluation

Five distinct models were trained for separate predictions. Overall, the machine learning models performed better in predicting functional outcomes, as measured by the mRS-90, compared to its performance in predicting mortality. Specifically, the Random Forest (RF) model demonstrated an impressive F1-Score of 0.815, indicating a strong balance between precision and recall. It also achieved a very good AUC of 0.871 and a low log loss of 0.140, underscoring its high predictive accuracy.

Conversely, when predicting mortality, the Logistic Regression (LR) model showed reasonable accuracy and discrimination power (AUC) of 0.819 and 0.859, respectively. However, it had a moderate F1-score of 0.44, indicating suboptimal precision and recall. This suggests that the inclusion of crucial predictive variables may need improvement.

**Fig. 2:**
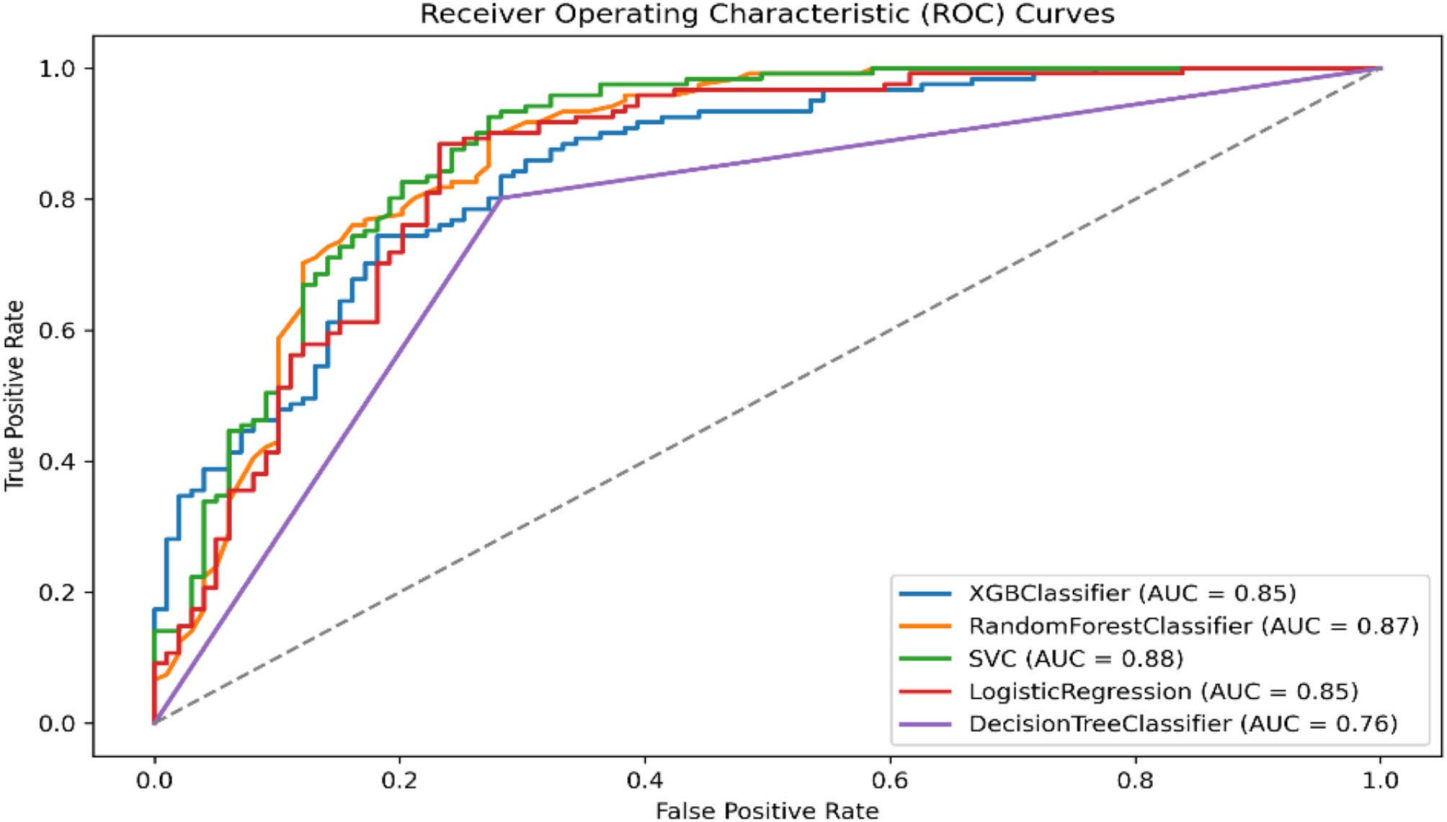
Area Under the Curve (AUC) for functional outcome (mRS-90)- All models.

**Fig. 3:**
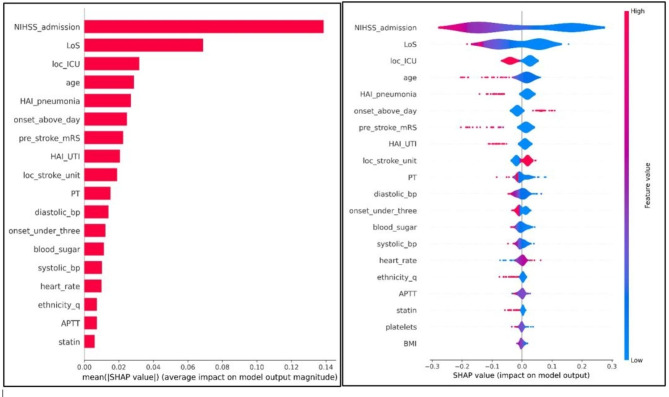
Area Under the Curve (AUC) for In-hospital mortality - All models.

In summary, based on the various evaluation metrics, we selected the Random Forest (RF) model for functional outcome prediction (mRS-90) and the Logistic Regression (LR) model for predicting in-hospital mortality as the primary choices for further SHAP analysis. Table 2 detail the models’ performance metrics. Figures 2 and 3 illustrate the discrimination power for the two selected models.

**Table 2:** Models performance for predicting functional outcome (mRS-90) and predicting in-hospital mortality.

Model	Accuracy	Precision	Specificity	Recall	F1-Score	AUC	MCC	Balanced Accuracy	Log Loss	Cohen’s Kappa	Gini Coefficient	Brier Score
*A: models performance for predicting functional outcome (mRS-90)*												
RF	0.791	0.795	0.737	0.835	0.815	0.871	0.576	0.786	0.440	0.575	0.742	0.140
XGB	0.777	0.777	0.707	0.835	0.805	0.850	0.548	0.771	0.605	0.546	0.699	0.173
SVM	0.782	0.835	0.818	0.752	0.791	0.882	0.567	0.785	0.443	0.564	0.764	0.141
DT	0.764	0.776	0.717	0.802	0.789	0.759	0.521	0.759	8.519	0.521	0.519	0.236
LR	0.759	0.815	0.798	0.727	0.769	0.854	0.523	0.763	0.492	0.519	0.708	0.157
*B: models performance for predicting in-hospital mortality*												
LR	0.819	0.304	0.818	0.828	0.444	0.859	0.428	0.823	0.415	0.363	0.717	0.133
SVM	0.807	0.284	0.809	0.793	0.418	0.848	0.396	0.801	0.228	0.332	0.695	0.067
XGB	0.904	0.421	0.964	0.276	0.333	0.859	0.291	0.620	0.311	0.284	0.718	0.080
DT	0.861	0.185	0.927	0.172	0.179	0.550	0.103	0.550	4.994	0.103	0.100	0.139
RF	0.910	0.333	0.993	0.034	0.063	0.893	0.083	0.514	0.207	0.047	0.786	0.063

RF: Random Forest, XGB: XGBoost, SVM: Support Vector Machines, DT: Decision Tree, LR: Logistic Regression

### SHAP analysis

**Fig. 4:**
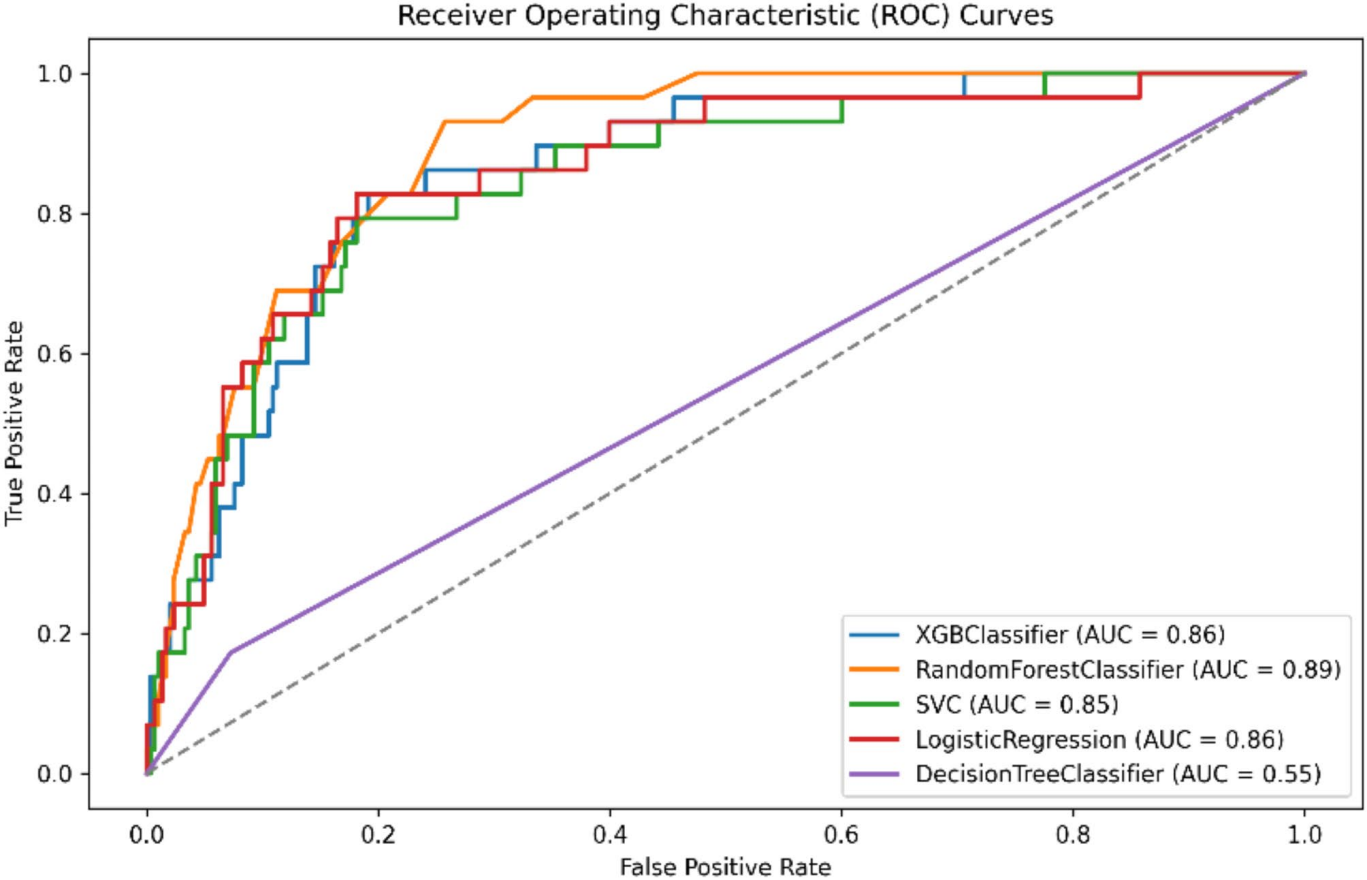
SHAP analysis for Random Forest model—Functional outcome (mRS-90).

**Fig. 5:**
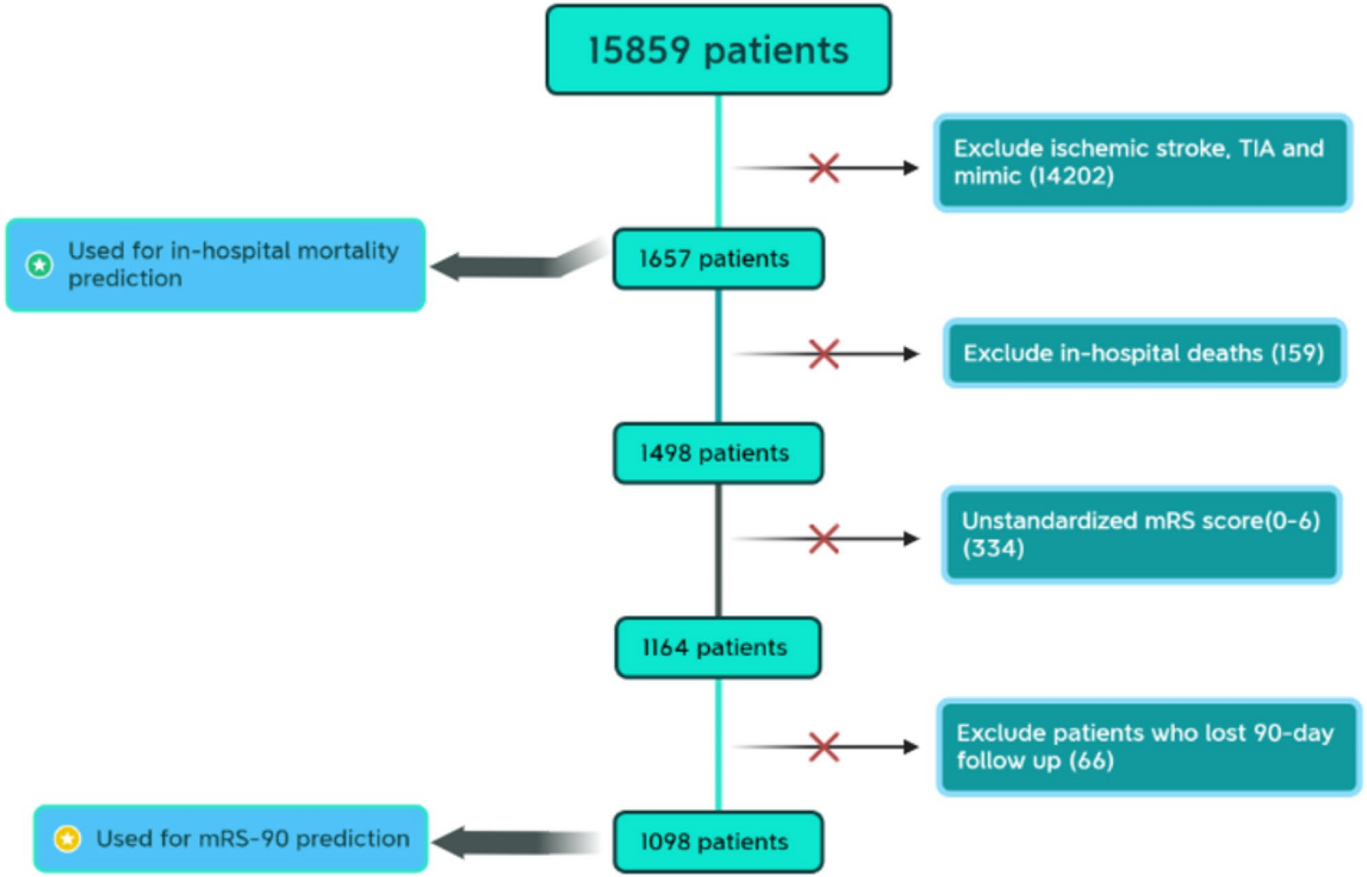
SHAPA analysis for Logistic Regression model—in-hospital mortality.

The SHAP analysis yielded invaluable insights into our predictive models for both mRS-90 and mortality. It revealed that, in descending order of importance for predicting mRS-90, the most influential factors were admission NIHSS, length of stay, admission location, age, and hospital-acquired pneumonia. In contrast, the primary predictors for mortality included NIHSS, admission location, ethnicity, blood sugar, HTN, and platelet count. For visual representations, refer to Figs. 4 and 5.

## Discussion

This study examined the effectiveness of five machine learning models and harnessed SHAP analysis to uncover the primary predictors for hemorrhagic stroke outcomes, specifically in-hospital mortality and the 90-day post-hospital discharge prognosis measured by mRS-90. The results underlined the superior performance of the RF model in predicting prognosis and the LR model in predicting in-hospital mortality, surpassing other models in accuracy. SHAP analysis on both models illuminated the pivotal variables that strengthened their predictive capabilities.

For predicting mRS-90, the influential variables included stroke severity (NIHSS upon admission), length of hospital stay (LOS), admission location, age, and hospital-acquired pneumonia. Conversely, for predicting in-hospital mortality in hemorrhagic stroke patients, the crucial factors encompassed NIHSS upon admission, admission location, patient ethnicity, blood sugar levels upon admission (RBS), hypertension (HTN), and admission platelet count. Remarkably, other variables exhibited lower SHAP values, indicating minimal contribution to the model’s predictive outcomes. Notably, except for LOS and hospital-acquired pneumonia, all these influential factors can be assessed early upon admission, making them valuable early indicators of prognosis. Consequently, this research can improve clinicians’ ability to promptly predict prognosis and mortality and customize care strategies to address mortality risk. Additionally, it can assist families in forming realistic expectations regarding anticipated outcomes, enhancing their involvement in the care delivery plan^17^.

### Predictors of hemorrhagic stroke prognosis (mRS-90)

Stroke severity, measured by the NIHSS, was identified as the most significant predictor of stroke prognosis, including mortality and functional disability across all timeframes^18–20^. In this study, the mean NIHSS score was 11.2 ± 8.6. Patients with unfavorable mRS-90 outcomes had significantly higher NIHSS scores than those with favorable outcomes (15.6 vs. 6.2, *p* < 0.05). This highlights the NIHSS’s critical role in early stroke outcome prediction and its importance in guiding personalized preventive care plans^21^.

The LOS plays a role in predicting stroke outcomes. Our study demonstrated that LOS significantly enhances the model’s predictive performance (Fig. 4). Generally, a longer LOS is often associated with in-hospital complications, such as hospital-acquired infections, which can potentially result in poor prognostic outcomes and may lead to mortality^22^. The mean LOS in our study was 11.2 ± 10.2 days. Approximately 80% of patients with LOS exceeding the mean value experienced unfavorable mRS-90 outcomes, whereas only 39% of those with a shorter LOS did so (*p*-value < 0.05). Additionally, the mean LOS for patients in the unfavorable outcome group was significantly longer than those with favorable mRS-90 outcomes (15 vs. 6.9 days, *p*-value < 0.05). It is important to note that LOS can be influenced by various factors, including stroke severity and in-hospital complications^23,24^. A secondary analysis revealed no statistically significant correlation between LOS and stroke severity (NIHSS). Still, it did find a significant association between LOS and the development of hospital-acquired infections, such as pneumonia (*p*-value < 0.05).

The choice of admission location significantly impacted stroke prognosis, aligning with previous studies suggesting that management in specialized stroke units can lead to reduced mortality, reduced length of stay, and improved patient outcomes^25,26^. Specifically, this research revealed that patients admitted to critical care units exhibited a higher incidence of unfavorable mRS-90 outcomes than those admitted to the stroke units or other units (such as general wards) (75.3% vs. 36% and 44.6%, respectively). This discrepancy can be attributed to the initial severity of stroke at admission, as measured by NIHSS, which necessitates closer monitoring and specialized care in critical care settings. Notably, patients admitted to critical care units had significantly higher mean NIHSS scores than those admitted to the stroke units or other units (16.7 vs. 7.3 and 7.2, respectively, *p*-value < 0.05).

Interestingly, although the mean NIHSS scores for patients admitted to stroke units and other units were statistically similar (7.3 vs. 7.2, *p*-value > 0.05), the unfavorable mRS-90 outcomes were notably higher for patients admitted to other units (44.6% vs. 36%, *p*-value < 0.05). This observation corroborates existing research indicating that stroke unit management improves outcomes, including reduced mortality and decreased dependence^25^. Evans and colleagues correlated that to the superior quality of specialized care delivered in the stroke units compared to the general wards, which includes superior monitoring and better management for fever, oxygenation, nutrition, and aspiration prevention^25^. Among patients admitted to the general wards, 5.4% developed hospital-acquired pneumonia, and 7.1% developed UTI compared to 4.3% and 4.5%, respectively, for those admitted to stroke units, corroborating the previous literature discussion about improved care outcomes.

This finding shed light on the need to address the capacity challenges in stroke units. When these specialized units cannot accommodate patients, individuals are often placed in general wards, which may provide a different level of care. Consequently, the study advocates exploring expanding stroke unit capacity to enhance patient outcomes, accelerate their recovery and return to the community, and ultimately improve their quality of life.

As observed in previous research, there is a well-documented association between age and an increased likelihood of unfavorable stroke outcomes^19^. Older individuals are known to be more susceptible to developing comorbidities that contribute to adverse outcomes and are linked to poorer disease prognosis^27^. Our study’s mean age was 51.3 ± 13 years, which could be considered relatively lower when assessed in regional and global scales^28^. Our supplementary analysis revealed that 56.5% of patients older than the mean age experienced unfavorable mRS-90 outcomes, compared to 50.6% of others (*p*-value < 0.05). Notably, the mean age of patients with unfavorable mRS-90 outcomes was significantly higher than those with favorable mRS-90 outcomes (51.12 vs. 48.8) with a *p*-value < 0.05. This finding emphasizes the importance of clinicians considering personalized preventive measures for older patients presenting with hemorrhagic stroke.

Additionally, the occurrence of hospital-acquired pneumonia emerged as a robust predictor of stroke patient prognosis. Stroke patients are known to be particularly vulnerable to infections during their hospital stay, which can significantly worsen their functional outcomes^29^. The study findings indicated that approximately 90% of patients who contracted hospital-acquired pneumonia experienced unfavorable mRS-90 outcomes, compared to 46% of those who did not develop hospital-acquired pneumonia (*p*-value < 0.05). This stresses the importance for clinicians to pay heightened attention to implementing evidence-based preventive measures, such as regular oral care, postural adjustments to prevent aspiration, dysphagia screening, and other strategies outlined by Grossmann and colleagues^30^.

### Predictors of the hemorrhagic stroke in-hospital mortality

Mortality is inherently regarded as an unfavorable outcome in the context of mRS. Consequently, it is logical to anticipate that factors contributing to a poor prognosis, as assessed by mRS-90, may overlap with those influencing mortality, such as NIHSS, in this study. The logistic regression (LR) model pinpointed stroke severity (NIHSS), admission location, patient ethnicity, blood sugar levels at admission (RBS), history of hypertension (HTN), and platelet count at admission as the primary predictors for in-hospital mortality in cases of hemorrhagic stroke.”

Like its significance in predicting mRS-90 outcomes, NIHSS emerges as a pivotal predictor of mortality. In our study, the mean NIHSS score stood at 12 ± 8.8. Notably, patients who deceased post hemorrhagic stroke exhibited a significantly higher mean NIHSS score compared to those who survived (21.3 vs. 11, *p*-value < 0.05). This sheds light on the importance of the severity of presentation following a hemorrhagic stroke event as an early indicator of adverse outcomes, prompting clinicians to consider early personalized preventive interventions^31^.

The location of admission significantly impacts stroke prognosis and mortality rates. Turner et al. found that admission to a stroke unit is associated with lower mortality and a higher likelihood of being discharged home within a year^26^. Patients in critical care units had a hospital mortality rate of 15%, while those in stroke units had rates above 0.7%, and other units saw a rate of 20.3% (*p*-value < 0.05). Despite lower severity scores (NIHSS 9.9 vs. 17.1), patients in general wards had the highest mortality, suggesting critical care may offer more appropriate treatment. The NIHSS score for other units was higher than in stroke units (9.6 vs. 5.5, *p*-value < 0.05), indicating a need to expand stroke unit capacity. Literature supports that specialized stroke units improve outcomes, though patients in critical care generally face poorer prognoses^32^.

Consistent with prior research findings, this study reaffirms the role of ethnicity in influencing stroke outcomes. Notably, our study reveals that patients from South Asian ethnicity exhibit a lower in-hospital mortality rate compared to those from MENA and other South-Asian ethnicities (8.6% vs. 11.2%, *p*-value < 0.05). Conversely, Qatari patients exhibit the highest mortality rate at 11.2%. Importantly, it is worth highlighting that there is no significant difference in NIHSS scores among the five ethnic groups. However, it is noteworthy that Qatari patients have the highest mean age, 65 years, compared to 59, 47, 47, and 50 years for MENA, South Asian, South-East Asian, and other ethnicities, respectively. In a broader context, this phenomenon can be understood by considering the demographic composition of Qatar. The Qatari population mirrors global age distribution patterns, reflecting a typical society. However, the situation changes when we shift our focus to expatriates. Among expatriates, there is a noticeable difference in age distribution. Most expatriates are younger and of working age, primarily residing in Qatar for employment purposes. This may explain why the mean age of Qatari patients is 64.9 ± 15 years compared to 49 ± 11 years for non-Qatari patients.

The fourth element that boosted the model’s predictive precision is the initial Random Blood Sugar (RBS) level. This study aligns with earlier research by establishing a connection between elevated RBS levels upon admission and an adverse prognosis^33,34^. It is reported that acute hyperglycemia increases brain damage, enlarges infarct sizes, and reduces cerebral blood volume. It also worsens stroke effects by intensifying reperfusion injury, oxidative stress, and inflammation. Further, acute hyperglycemia increases the risk of platelet aggregation, complicating recovery^35,36^. In our study, the mean RBS level measured 8.8 ± 4 mmol/l, and notably, the mean RBS level among patients who did not survive their hospital stay was significantly higher than that of the group that did survive (10.6 vs. 8.6, with a *p*-value < 0.05).

This study revealed a paradox where patients with a known history of HTN had significantly lower in-hospital mortality rates following hemorrhagic stroke compared to those without a prior HTN diagnosis (7.1% vs. 22.1%, *p* < 0.05), contradicting previous literature^37^. Patients with HTN also had lower NIHSS scores, which might be due to regular antihypertensive treatment reducing stroke severity. This could be linked to the idea that systolic blood pressure variability increases stroke mortality risk^38,39^, with antihypertensive medications reducing these fluctuations. The study’s limitations, including missing data on the hemorrhage site and size, suggest further research to understand these findings fully.

Previous studies have established a notable inverse relationship between hemorrhagic stroke mortality and mean platelet count^40,41^. In accordance with this existing literature, our study underscores the potential of a lower platelet count as an early indicator of hemorrhagic stroke mortality. Our secondary analysis unveiled that the mean platelet counts among patients who did not survive was significantly lower than that of the group that did survive (248.6 vs. 259.3, *p*-value < 0.05).

This study has revealed an intriguing finding: the severity of the initial presentation in cases of hemorrhagic stroke is a paramount predictor for prognosis and can potentially function as an early indicator. Among all the factors considered, the NIHSS exhibited the highest mean SHAP value (refer to Figs. 4 and 5). Importantly, NIHSS and admission location emerged as shared predictors for hemorrhagic stroke outcomes, demonstrating their potential as early indicators to guide clinicians in devising preventive care plans.

As we look to the future, it becomes evident that thorough validation of the study’s prediction models is essential to demonstrate their utility in clinical decision-making. Despite the inherent limitations, this research holds promise by shedding light on critical areas for improvement. Notably, it underlines the importance of enhancing the stroke registry by incorporating vital variables such as hemorrhage size, location, and imaging data. Furthermore, this study may pave the way for the development of localized predictive models, facilitating early prognostication, which are crucial elements in creating personalized care plans and supporting the endeavors of precision medicine.

### Limitations

This study presents several notable limitations that warrant acknowledgment. Firstly, it relies on retrospective data from a solitary medical center, potentially introducing bias to selection and constraining the generalizability of findings to broader populations. Furthermore, the absence of crucial variables in the stroke registry, including hemorrhage size, location, imaging data, and socioeconomic factors, limits the depth of analysis and overlooks potential influential factors in stroke outcomes.

Utilizing historical data may only partially encompass evolving treatment strategies and shifting patient demographics, potentially affecting the model’s applicability in contemporary clinical contexts. Moreover, the prediction window for in-hospital mortality and the 90-day functional status measured by mRS may need to adequately capture longer-term outcomes, potentially limiting its utility in assessing extended prognostic trends.

Also, the dataset exhibits class imbalance, with a pronounced underrepresentation of mortality cases. This imbalance introduces complexity during model development and evaluation, potentially leading to biased model performance despite mitigation efforts. Consequently, it affects result clarity and raises concerns about the machine learning model’s utility in guiding clinical decision-making. While machine learning techniques were applied to predict hemorrhagic stroke outcomes, these models necessitate comprehensive external validation to confirm their robustness and applicability.

However, although the models exhibit promise, demonstrating moderate precision and recall rates, especially in predicting in-hospital mortality, there remains room for improvement. Utilizing machine learning models introduces inherent intricacies, potentially complicating their interpretation within clinical settings and integration into practical healthcare workflows.

Lastly, while SHAP analysis identifies critical predictors, this study does not establish definitive causal relationships between these variables and stroke mortality. Thus, while this research advances our understanding of stroke mortality prediction, it is essential to consider its implications within the context of these limitations. Despite these constraints, this study provides valuable insights into predicting hemorrhagic stroke outcomes. It underscores the importance of enhancing data collection within stroke registries to enhance predictive model accuracy and relevance. Further research using more extensive and diverse datasets is imperative to ensure the robustness and refinement of the presented predictive models.

## Conclusion

This study explores the prediction of hemorrhagic stroke outcomes using machine learning models and SHAP analysis to identify critical factors. Notably, the severity of hemorrhagic stroke at presentation is a crucial predictor of outcomes, including mortality. The study underscores the significant role of admission location in shaping stroke outcomes, emphasizing the impact of specialized and critical care units in providing superior care. This highlights the need for policy discussions on expanding stroke unit capacity to improve outcomes and reduce the economic burden of long-term disability. However, the study faces limitations, including its retrospective nature, single-center data reliance, and missing variables in the stroke registry, which introduces selection bias and limit generalizability. Additionally, the class imbalance in the dataset poses challenges in model development. Nevertheless, this research underlines improving data collection in stroke registries. It offers promises for more accurate predictive models for hemorrhagic stroke outcomes, mainly since it encompasses the entire population of patients seeking specialized care. Future research should prioritize larger, more diverse datasets and a deeper exploration of variables to improve clinical practice. Additionally, adopting a multi-centric approach in future studies will enhance generalizability and contribute to advancing personalized stroke care.

## Data Availability

The datasets generated and/or analyzed during the current study are available from the corresponding author on reasonable request and subject to appropriate ethical approvals.
